# Reframing Fixation Strategy in Total Knee Arthroplasty With Tibial Bone Density as a Central Criterion

**DOI:** 10.2106/JBJS.OA.26.00087

**Published:** 2026-06-25

**Authors:** Yoshinori Mikashima, Ken Okazaki

**Affiliations:** 1 Oume Knee Surgery Center, Takagi Hospital, Tokyo, Japan; 2 Department of Orthopaedics, Tokyo Women’s Medical University, Tokyo, Japan

## Abstract

**Background:**

Cementless total knee arthroplasty (TKA) has gained renewed interest with advances in implant design, porous coatings, and surgical accuracy. However, early aseptic loosening continues to be reported, particularly in patients with reduced tibial bone mineral density (BMD). Cemented TKA also demonstrates loosening related to thin or insufficient cement mantles, especially in younger, high-body mass index, or highly active patients. Despite advances in available fixation methods, clear and reproducible criteria to guide fixation selection have yet to be established.

**Methods:**

A narrative review was conducted to summarize recent evidence regarding fixation strategy in TKA with a focus on tibial BMD. A PubMed literature search identified studies published between 2016 and 2026 using terms related to fixation methods (cementless and cemented TKA), tibial bone density, loosening mechanisms (migration, subsidence, early failure), and cementing techniques (cement mantle thickness and cement penetration). The initial search yielded 289 articles, of which 84 studies relevant to bone density, fixation stability, implant design, and loosening mechanisms were included.

**Results:**

Large database studies consistently reported higher early aseptic loosening rates in cementless TKA, particularly in patients with low tibial BMD. Radiostereometric analyses demonstrated increased migration of cementless tibial components in low-density bone. In cemented TKA, inadequate cement penetration and thin or nonuniform cement mantles were strongly associated with tibial loosening. Recent computed tomography–based techniques—including hydroxyapatite-equivalent BMD and dual-energy computed tomography volumetric BMD—show strong correlations with trabecular bone strength and provide practical thresholds for fixation selection.

**Conclusion:**

Based on the results of all the papers reviewed, tibial BMD values are important and do influence the loosening rate of tibial components. Those patients with tibial BMD values below 46.4 HA/cm^3^ support the use of cemented fixation, while those exceeding 78.4 HA/cm^3^ seem to be better candidates for cementless fixation due to limited cement penetration.

## Introduction

Cementless total knee arthroplasty (TKA) has re-emerged as an important surgical option in contemporary arthroplasty practice, supported by advances in implant design, porous coatings, three-dimensional printing technology, and improved surgical accuracy^1-3^. These developments have enhanced operative efficiency and biological fixation, leading to a global increase in the use of cementless TKA^4,5^. However, early loosening of cementless TKA has continued to be reported in recent years, and concerns regarding its initial stability remain unresolved^6,7^.

This raises an important question: Is cemented TKA truly a definitive solution? Recent large-scale registry analyses have reported no significant difference in aseptic loosening between cemented and cementless fixation, with odds ratios ranging from 0.67 to 1.09 across 90-day to 2-year follow-up periods^8^. Cemented fixation demonstrates a lower incidence of early loosening compared with cementless fixation^9^. However, higher rates of loosening have been observed in the mid-term to long-term follow-up of cemented fixation^9^. In particular, thin cement mantles and insufficient cement penetration have been identified as major contributors to tibial loosening, resulting in increased revision rates^10,11^. In addition, tibial bone density has been shown to influence cement penetration and cement mantle thickness, as demonstrated in our randomized controlled trial^12^.

Despite technological progress, failures continue to occur with both fixation methods. A major underlying reason is that clear and reproducible patient selection criteria have not yet been established for either cemented or cementless fixation^13^. In clinical practice, there is a growing need for simple, reliable, and easily applicable criteria to guide fixation choice. Understanding the mechanisms of failure in both cemented and cementless TKA, together with quantitative assessment of tibial bone mineral density (BMD), may offer a rational basis for selecting fixation^12,14^. Accordingly, we conducted a narrative review with a focus on a bone density-driven approach to fixation strategy.

## Methods

This narrative review summarizes the latest evidence regarding patient selection for cementless TKA. A literature search was conducted in the MEDLINE database (PubMed) for studies published between January 1, 2016, and December 31, 2025. The search strategy used the following Boolean operators: (“total knee arthroplasty” OR “TKA”) AND (cementless OR uncemented OR cemented) AND (tibial OR tibia) AND (loosening OR migration OR subsidence OR “early failure” OR “aseptic loosening”) AND (bone OR “bone density” OR BMD OR osteoporosis OR “bone quality” OR “CT determined density”). Only articles published in English were included. A total of 289 articles were retrieved through the PubMed search, and 2 additional relevant studies were identified through a manual search. Of these, 84 publications that met the conceptual criteria for fixation strategy and bone density assessment were included in the final review.

Particular emphasis was placed on recent reviews addressing indications for cementless fixation^5,6^. Computed tomography (CT)–based methods for assessing bone density^14-16^, the relationship between bone density and initial fixation stability^5,17^ and studies concerning cement mantle thickness and cement penetration.^10-12^

## Aseptic Loosening in Cementless TKA

### Early Aseptic Loosening Reported in Large Database Studies of Cementless TKA

Large database studies have reported early aseptic loosening in cementless TKA (Table I)^7^. Chiou et al. analyzed more than 320,000 cases and found that single-component revision within 1 year was significantly more frequent in the cementless cohort^7^. Forlenza et al. also demonstrated that the risk of revision due to aseptic loosening within 2 years was approximately 2.3 times higher in cementless TKA compared with cemented fixation^18^. Similarly, Andronic et al. reported that 7% of patients required revision for aseptic loosening at a mean of 10.4 months postoperatively^19^. Despite recent technological advances, early aseptic loosening in cementless TKA remains a serious concern.

**TABLE I:** Recent Reports of Early Failure in Cementless Total Knee Arthroplasty

Author (Ref#)	Study Design	Yr	Summary
Andronic O^19^	Cohort study	2025	A total of 9 knees (7%) from the cementless cohort underwent revision surgery, all due to aseptic loosening of the baseplate at a mean follow-up of 10.4 mo.
Haslhofer A	Biomechanical study	2025	Low trabecular density increased micromotion and impaired osseointegration
Agarwal A	Nationwide database	2024	Cementless TKA use increased, but early revision risk remained higher in selected subgroups
Deans V	Systematic review	2024	Osteopenia/osteoporosis significantly increased the risk of early failure in cementless TKA
Nam D^23^	Registry analysis	2024	Cementless TKA demonstrated the greatest overall revision rate at the 5-yr follow-up
Gibian JT^22^	Case series	2024	Early catastrophic failures of a two-peg cementless tibial baseplate design attributed to inadequate metaphyseal support
Chiou D^7^	Database study	2023	Cementless TKAs more likely than cemented TKAs to require revisions at both 90 d (0.39% vs. 0.21%) and 1 yr (0.87% vs. 0.45%)
Forlenza EM^18^	Database study	2023	Increased risk of revision for aseptic loosening in cementless TKA compared with cemented TKA at 2 yrs postoperatively (OR 2.34, P < 0.001)
Roth JD	Registry analysis	2023	Higher early revision rates in cementless TKA among patients with osteoporosis or osteopenia
Lachiewicz PF	Review	2022	Early loosening remains a concern in cementless TKA, especially in low-BMD patients
			Early tibial subsidence more common in patients with low tibial BMD
Sappey-Marinier E	RSA study	2022	Increased early migration in cementless tibial components in patients with low trabecular density
Hernandez NM	Cohort study	2021	Early tibial component subsidence in cementless TKA associated with small tibial metaphyseal flare
Goh GS	Cohort study	2021	Early tibial loosening linked to inadequate metaphyseal engagement in certain designs
Klasan A^5^	Systematic review	2021	Early loosening in cementless TKA linked to bone quality and tibial morphology
Goh GS	Prospective cohort	2020	Low proximal tibial BMD predicted early tibial subsidence in cementless TKA
Meneghini RM^6^	Multicenter cohort	2020	Early tibial loosening occurred predominantly in elderly women with osteoporotic bone
Abdel MP	Institutional cohort	2020	Higher early revision rates observed in cementless TKA among elderly females
Kang KT	Finite element analysis	2020	Low-density tibial bone increases micromotion and risk of early loosening in cementless TKA
Kamath AF	Registry study	2019	Cementless tibial components showed increased early revision risk compared with cemented fixation
Huang R	Multicenter cohort	2019	Early tibial loosening found in cementless TKA among elderly Asian women with low metaphyseal density
Vertullo CJ	Australian registry	2018	Cementless fixation showed higher early migration in patients with small tibial metaphysis
Song SJ	Cohort study	2018	Early aseptic loosening associated with undersized tibial components in cementless TKA
Parratte S	Cohort study	2018	Cementless tibial components had higher early migration but stabilized by 1 yr in younger patients
Andersen MR^21^	RSA study	2017	Low preoperative BMD is related to high migration of tibia components in uncemented TKA
Berend ME	Case series	2017	Early catastrophic tibial failures linked to insufficient metaphyseal support
Watters TS	Institutional cohort	2017	Early aseptic loosening occurred in cementless tibial components implanted in patients with low bone quality
Haughom BD	Database study	2016	Early failure risk increased in cementless TKA among patients with low BMI or low bone density
Bagsby DT	Case series	2016	Described early tibial tray failures in cementless TKA linked to insufficient initial fixation

Studies are organized in reverse chronological order.

BMD = bone mineral density, BMI = body mass index, DECT = dual-energy computed tomography, OR = odds ratio, RSA = radiostereometric analysis, and TKA = total knee arthroplasty.

These studies further indicated that compromised bone quality is a key contributing factor to early aseptic loosening in cementless TKA. Reduced bone quality was associated with a higher risk of early loosening^18^, while poor bone quality, high body mass index (BMI), and high activity levels were linked to insufficient initial fixation^7^. Moreover, a subgroup experiencing early loosening consisted predominantly of older female patients, suggesting an underlying role of osteoporosis.^19^

### Mechanism of Early Loosening in Cementless TKA

Early aseptic loosening in cementless TKA is believed to occur when adequate press-fit fixation cannot be achieved in tibiae with reduced BMD^5^. Insufficient primary stability increases micromotion at the implant–bone interface, and sustained micromotion exceeding approximately 150 μm has been shown to inhibit osteoblastic bone formation and instead promote the ingrowth of fibrous tissue^20^. Such fibrous ingrowth prevents the establishment of osseointegration, which is essential for cementless fixation, ultimately leading to early aseptic loosening^20^. Supporting this mechanism in a clinical context, Andersen et al. used radiostereometric analysis to demonstrate that a lower preoperative BMD was associated with increased migration of cementless tibial components.^21^

### Design-Related Early Loosening in Cementless TKA

Early aseptic loosening in cementless TKA is influenced not only by patient factors and bone quality but also by design-specific characteristics of the implant. A representative example is the two-peg tibial baseplate, for which several reports have described a higher incidence of early loosening^22^. By contrast, recent implant designs incorporating a central keel combined with a four-peg configuration have been reported to improve primary stability by reducing rotational micromotion^1,23,24^. These findings indicate that the success of cementless TKA depends not only on adequate bone quality but also on implant designs capable of achieving sufficient initial fixation.

## Aseptic Loosening in Cemented TKA

### Aseptic Loosening Reported in Large Database Studies of Cemented TKA

Large database studies have consistently reported a measurable incidence of aseptic loosening in cemented TKA (Table II)^25-27^. Notably, higher rates of loosening have been observed in younger, high-BMI, and highly active patients, in whom cemented TKA demonstrates a greater risk of failure compared with cementless fixation.^25-27^

**TABLE II:** Key Studies Reporting Loosening in Cemented TKA Related to Cement Mantle or Cement Penetration

Author (Ref#)	Study Design	Yr	Summary
Mikashima Y^21^	Comparative clinical study	2026	Identified tibial BMD as the key determinant of cement mantle thickness, with high BMD reducing penetration
Schaffler BC^11^	Case–Control Study	2025	Identified thin or nonuniform cement mantles as an independent risk factor for aseptic tibial loosening in cemented TKA
Yao K^25^	Systematic review	2025	Identified risk factors include younger age, high BMI, male sex, high activity level, and technical factors such as inadequate cement mantle or penetration
Ayati FM^26^	Retrospective cohort study	2025	Long-term cohort study demonstrating that elevated BMI is associated with a higher rate of aseptic loosening in primary TKA
van Duren BH^27^	Comparative cohort study	2025	Obese patients had higher loosening rates with unstemmed cemented tibial components than with uncemented or stemmed designs
Ito T	Retrospective radiographic study	2024	Showed that insufficient cement penetration correlated with radiolucent line formation around the tibial component
Thompson N	Narrative review	2024	Summarized mechanisms of aseptic loosening in cemented TKA, emphasizing cement–bone interface failure and inadequate cement interdigitation
Reid D	Educational review	2024	Highlighted cement mantle defects and insufficient pressurization as major contributors to tibial aseptic loosening
Orthopaedics & Trauma Review	Narrative review	2024	Reaffirmed that cement mantle quality is central to preventing tibial loosening
J ISAKOS Group	Retrospective radiographic study	2024	Demonstrated that poor cement penetration predicts radiolucent lines and early tibial fixation loss
Kobayashi H	Case–control study	2023	Identified thin or nonuniform cement mantles as an independent risk factor for early tibial loosening after cemented TKA
van Otten TJM	Narrative review	2023	Summarized early tibial loosening at the cement–implant interface, highlighting mantle defects
Brown M	Technical review	2022	Emphasized that thin cement mantles and inadequate cement penetration remain key modifiable risk factors for tibial failure
Australian Orthopaedic Association NJR^9^	Registry analysis	2021	Demonstrated that cemented TKA still shows measurable tibial loosening, partly due to variability in cementing technique
Martin JR	Case–control	2021	Identified thin cement mantles as a cause of tibial loosening in early failures
Cox ZC	Radiographic cohort	2021	Demonstrated that cement mantle thickness strongly correlates with tibial aseptic loosening
van der Lelij A^3^	RSA clinical study	2020	Showed that thin or poor-quality cement mantles are associated with increased tibial micromotion
Swedish Knee Arthroplasty Register^8^	Registry report	2020	Reported persistent tibial loosening in cemented TKA; cement technique cited as modifiable factor
Johansson B	RSA migration study	2019	Found that early radiolucent lines due to poor cement interdigitation predicted later tibial component loosening
Australian Orthopaedic Association NJR^9^	Registry analysis	2019	Demonstrated that cemented TKA still shows tibial loosening, partly due to cementation technique variability
Smith L	Multicenter cohort study	2018	Reported that cement viscosity, timing, and pressurization technique significantly influenced tibial fixation and risk of early loosening
Berend ME	Multicenter cohort	2018	Identified inadequate cement penetration as a major factor in early tibial component failure
Abdel MP	Radiographic cohort	2017	Reported that radiolucent lines at the cement–bone interface predict later tibial loosening
Ritter MA	Clinical cohort study	2016	Demonstrated that insufficient cement interdigitation increases the risk of tibial loosening in cemented TKA

BMD = bone mineral density, BMI = body mass index, CT = computed tomography, DECT = dual-energy computed tomography, ISAKOS = International Society of Arthroscopy, Knee Surgery and Orthopaedic Sports Medicine, NJR = National Joint Registry, RSA = radiostereometric analysis, and TKA = total knee arthroplasty.

### Mechanisms of Loosening in Cemented TKA

The primary mechanism of aseptic loosening in cemented TKA is thought to involve the compressive failure or fracture of a thin cement mantle, which compromises the mechanical integrity of the fixation interface^10,11,28^. In addition, nonuniformity of the cement mantle can reduce interfacial strength and increase micromotion. Patients with high BMI and activity levels tend to exhibit increased proximal tibial bone density, which reduces cement penetration and limits adequate interdigitation^29^. Under such conditions, the resulting cement layer becomes mechanically vulnerable and more prone to collapse under repetitive loading, providing a plausible explanation for the higher loosening rates observed in this population.

## Bone-Density–Driven Preoperative Strategy

**Fig. 1:**
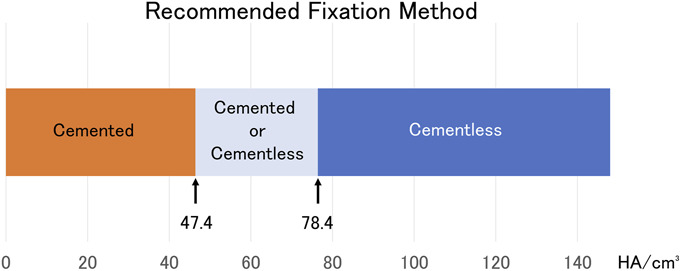
Recommended fixation method according to tibial BMD (HA/cm^3^). Cemented fixation is suggested for BMD ≤ 46.4, either method for 46.4 to 78.4, and cementless fixation for ≥ 78.4. BMD = bone mineral density.

Evaluating tibial BMD at the planned resection level during preoperative planning is essential for selecting a fixation method that minimizes the risk of failure (Table III)^12,14^. Our recent work demonstrated a strong correlation between the hydroxyapatite (HA) values at the tibial resection level and the actual trabecular bone strength, expressed as the intraoperative cancellous bone durability^14^. We also reported that a tibial BMD of 46.4 HA/cm^3^ or lower indicates insufficient bone quality, making cemented fixation the more appropriate option (Fig. 1)^14^. Furthermore, knees with low tibial BMD tend to allow the formation of a uniform and adequately thick cement mantle^12^. Conversely, in knees with a tibial BMD of 76.4 HA/cm^3^ or higher, cement penetration is limited, and a cement mantle thickness greater than 2.1 mm cannot be reliably achieved^12^. In such high-density tibiae, cementless fixation may represent a rational choice (Fig. 1). However, this interpretation has not yet been clinically proven to increase loosening risk in cemented fixation. Therefore, it should be regarded as a hypothesis that requires confirmation in future clinical studies. If cemented fixation is selected in these cases, additional drilling perforations can serve as an appropriate strategy to enhance cement interdigitation.^12,30^ By contrast, tibia with intermediate BMD values (46.4-76.4 HA/cm^3^) may be suitable for either cemented or cementless fixation, as both methods can achieve adequate initial stability within this range (Fig. 1).^12^

**TABLE III:** Key Literature on Bone Density Assessment Related to Total Knee Arthroplasty

Author	Assessment Method	Yr	Summary
Mikashima Y^14^	CT (HA-equivalent BMD)	2025	First study to correlate resection-level HA density with ICBD
Lee JK	DECT-based study	2025	Proposed 45.5 mg/cm^3^ as a safety threshold for cementless TKA using DECT-derived vBMD
Lin YC	DECT-based study	2025	Demonstrated strong correlation between DECT vBMD and mechanical bone strength
Deans VM	DEXA	2024	Systematic review linking osteopenia/osteoporosis to early failure in cementless TKA
Okuno Y	CT (HU)	2024	Using CT-derived HU to quantify tibial bone density, the study showed a negative correlation between HU values and cement penetration depth
Ito J^40^	QCT	2023	Developed QCT-based method to estimate bone density from plain radiographs
Schröder HM	RSA	2020	Longitudinal RSA series showing how bone quality affects early migration and long-term fixation
Andersen MR^21^	DEXA	2017	Showed low preoperative BMD increases tibial component migration in cementless TKA (RSA)
Winther NS	RSA	2016	Longitudinal RSA series showing how bone quality affects early migration and long-term fixation

CT = computed tomography, DECT = dual-energy computed tomography, DEXA = dual-energy x-ray absorptiometry, HA = hydroxyapatite, HU = Hounsfield units, ICBD = intraoperative cancellous bone durability, QCT = quantitative computed tomography, RSA = radiostereometric analysis, TKA = total knee arthroplasty, and vBMD = volumetric bone mineral density.

Tibial BMD has been shown to correlate more closely with the actual cancellous bone strength of the proximal tibia than hip dual-energy x-ray absorptiometry (DEXA) measurements^14^. Therefore, CT-based assessment may better reflect local bone quality relevant to tibial fixation. Previous studies have also reported sex-specific differences in the relationship between proximal tibial BMD and hip DEXA values^31^. A strong correlation has been observed in men, whereas the correlation is much weaker in women. These findings highlight the limitations of relying solely on hip DEXA to evaluate tibial bone quality.

At our institution, preoperative CT scanning is routinely performed as part of the standard preoperative evaluation for all TKA candidates to assess the underlying pathology. During this examination, tibial bone density at the planned resection level is simultaneously measured. The cost of a knee CT scan is approximately 110 USD in Japan, and it is covered by the national health insurance system^32^. However, some countries use bundled payment models. Even in such settings, CT remains an important examination that can influence the choice of fixation method in TKA. Therefore, we believe that its use represents a cost-effective diagnostic modality.

The radiation dose for knee CT was an average effective dose of 0.034 mSv^33^. This corresponds to approximately 3 times the radiation dose of a chest radiograph, which is still considered a low level of exposure.^34^

## Assessment of CT-Based Tibial Bone Density

### Hounsfield Unit

Hounsfield units (HU) represent a numerical index of radiographic brightness on CT and are widely used as a surrogate for estimating bone density. However, HU values are highly susceptible to variations in scanning parameters and differences among CT scanners, resulting in substantial interinstitutional variability.^35,36^

### HA-Equivalent BMD

**Fig. 2:**
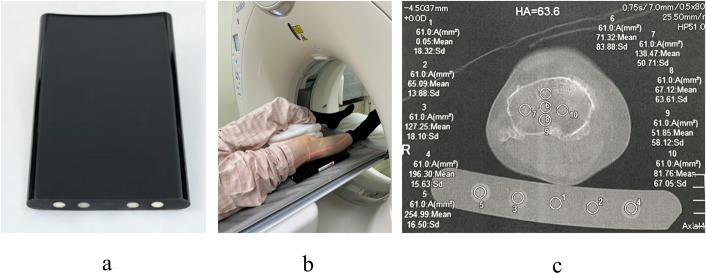
**Fig. 2-A** CT-based bone density quantification Phantom. The Phantom contains five 15-mm cylindrical inserts corresponding to 0, 50, 100, 150, and 200 mg HA/cm3. **Fig. 2-B** A Phantom was placed under the knee to be operated during the scan. **Fig. 2-C** HA indicates the hydroxyapatite value. CT = computed tomography.

The HA value is a more accurate indicator of BMD, obtained by converting HU values into HA-equivalent concentrations. This conversion requires simultaneous scanning of a calibration phantom with known mineral densities, which corrects for scanner-dependent variability. Because phantom calibration enables cross-institutional comparability, HA values are more reliable than HU values and are useful for determining the suitability of cementless TKA^14^. The calibration phantom used for HA measurement is compact and can be easily placed beneath the knee during scanning (Figs. 2-A, 2-B, and 2-C).^14^

### Dual-Energy CT Volumetric BMD

Dual-Energy CT Volumetric BMD (DECT vBMD) is a true volumetric bone density metric that directly estimates mineral content using 2 different x-ray energy spectra. Unlike conventional CT, in which HU values are strongly influenced by scanning conditions, dual-energy computed tomography leverages material-specific attenuation characteristics, allowing more accurate quantification of bone mineral content^15,16,37^. DECT vBMD does not require phantom calibration and is less affected by differences among CT scanners, making it a physically stable and increasingly recognized method for bone density assessment^15,16,37^. Recent studies have demonstrated a strong correlation between DECT vBMD and actual bone strength, and Lee et al. proposed 45.5 mg/cm^3^ as a safety threshold for cementless TKA.^15^

### Modern Surgical Technology

Advances in robotic and navigation technologies have substantially contributed to securing primary stability in cementless TKA by enabling highly accurate bone resections^38,39^. By improving the precision of bone cuts and implant positioning, these systems enhance conformity at the implant–bone interface, which may in turn reduce micromotion. Moreover, in cases with considerable anatomical variability of the tibia or in patients with low BMD, these technologies allow intraoperative adjustments that accurately reflect individual morphology^38,40^. This capability improves safety and reproducibility.

Conversely, several studies have reported that experienced knee surgeons are capable of achieving high accuracy in bone resection and implant placement even without robotic assistance^41,42^. Thus, in the hands of skilled surgeons, conventional techniques may still provide sufficiently stable press-fit fixation to allow cementless implantation to be performed safely.

### Future Directions

Recent advances have introduced methods for estimating BMD from plain radiographs using dedicated applications^31^. With the continued development of artificial intelligence-based image analysis, it is likely that techniques capable of automatically estimating tibial BMD and trabecular architecture from CT or radiographic images will become clinically feasible^43^. Furthermore, integrating risk-prediction models that incorporate bone density, tibial morphology, and patient-specific factors into preoperative planning software linked to robotic or navigation systems may enable more accurate and individualized decision making^44^. On the implant side, the development of “personalized designs” that optimize press-fit strength and porous architecture according to patient-specific bone quality is also anticipated^45^. Integration of these technologies may enable a more evidence-based standardization of indications for cementless TKA and reduce variability among surgeons.

## Conclusion

Selecting the fixation method based primarily on knee bone quality, particularly tibial BMD, represents a rational strategy to enhance both the safety of TKA and the advantages inherent to each fixation approach. The development of a simple, universally applicable method for assessing knee bone density is anticipated in the future.

## Funding

No benefits in any form have been received or will be received from a commercial party related directly or indirectly to the subject of this article.

## Ethical Approval

Not applicable. This study is a narrative review of previously published literature and did not involve human subjects or identifiable patient data.

## Informed Consent

Not applicable. No new human data were collected for this review.

## References

[1] Mikashima Y Imamura H Yano K Ikari K Takagi H Okazaki K. Low frequency of radiolucent lines in 3D-printed porous-coated tibial cementless baseplate at 1-year follow-up. J Exp Orthop. 2025;12(3):e70356. 40655261 10.1002/jeo2.70356 PMC12255930

[2] Zappley NR Restrepo S Fraval A Ong AC Hozack W. 10-year follow-up for a new three-dimensional printed cementless total knee arthroplasty. J Arthroplasty. 2025;40(7S1):S250-S255. 10.1016/j.arth.2025.02.043 40121147

[3] Van Der Lelij TJN Marang-Van De Mheen PJ Kaptein BL Toksvig-Larsen S Nelissen RGHH. Continued stabilization of a cementless 3D-Printed total knee arthroplasty: five-year results of a randomized controlled trial using radiostereometric analysis. J Bone Joint Surg. 2023;105(21):1686-94. 37651549 10.2106/JBJS.23.00221 PMC10609712

[4] de Grae MNM Nasehi A Dalury DF Masri BA Sheridan GA. Improved performance of cementless total knee arthroplasty (TKA)across international registries: a comparative review. Ir J Med Sci. 2025;194(2):675-81. 39928234 10.1007/s11845-025-03888-6 PMC12031968

[5] Haslhofer DJ Kraml N Stadler C Gotterbarm T Klotz MC Klasan A. Cementless fixation in total knee arthroplasty: current evidence and future perspective. Arch Orthop Trauma Surg. 2024;145(1):101. 39731597 10.1007/s00402-024-05670-2 PMC11682007

[6] Mosher ZA Bolognesi MP Malkani AL Meneghini RM Oni JK Fricka KB. Cementless total knee arthroplasty: a resurgence—who, when, where, and how? J Arthroplasty. 2024;39(9S2):S45-S53. 38458333 10.1016/j.arth.2024.02.078

[7] Chiou D Li AK Upfill-Brown A Arshi A Hsiue P Chen K Stavrakis A Photopoulos CD. Cementless compared to cemented total knee arthroplasty is associated with more revisions within 1 year of index surgery. Arthroplast Today. 2023;21:101122. 37521088 10.1016/j.artd.2023.101122 PMC10382689

[8] Monarrez R Dubin J Bains SS Hameed D Moore MC Chen Z Mont MA Delanois RE Nace J. Cemented is not superior to cementless total knee arthroplasty for complications: a propensity score matched analysis. Eur J Orthop Surg Traumatol. 2024;34(4):1825-30. 38429555 10.1007/s00590-024-03847-4

[9] Swedish Knee Arthroplasty Register. Swedish Knee Arthroplasty Register – Annual Report 2019; 2019.

[10] Sasaki R Nagashima M Tanaka K Takeshima K. Relationship between cement penetration and incidence of a radiolucent line around the tibia 2 years after total knee arthroplasty: a retrospective study. J ISAKOS. 2024;9(4):609-14. 38825183 10.1016/j.jisako.2024.05.015

[11] Schaffler BC Robin JX Katzman J Arshi A Rozell JC Schwarzkopf R. Aseptic tibial loosening is associated with thickness of the cement: a radiographic case–control study. J Arthroplasty. 2025;40(7):1869-74. Published online December 2024. 39710212 10.1016/j.arth.2024.12.023

[12] Mikashima Y Imamura H Yano K Ikari K Takagi H Okazaki K. Cement mantle thickness in total knee arthroplasty more closely associated with tibial bone density than cement viscosity findings from a randomized controlled trial. Bone Joint Open. 2026;7(4):549-56. 41985891 10.1302/2633-1462.74.BJO-2025-0348.R2 PMC13082887

[13] Kamath AF Siddiqi A Malkani AL Krebs VE. Cementless fixation in primary total knee arthroplasty: historical perspective to contemporary application. J Am Acad Orthop Surg. 2021;29(8):e363-79. 33399290 10.5435/JAAOS-D-20-00569

[14] Mikashima Y Imamura H Yano K Ikari K Takagi H Okazaki K. Preoperative computer tomography scans can accurately evaluate tibial bone mineral density for selecting bone fixation in total knee arthroplasty. J ISAKOS. 2025;12:100894. 40334842 10.1016/j.jisako.2025.100894

[15] Lee DH Kwak DS Lee SW Kim YD Cho N Koh IJ. Volumetric bone mineral density assessed by dual-energy CT predicts bone strength suitability for cementless total knee arthroplasty. Medicina (Lithuania). 2025;61(7):1305. 10.3390/medicina61071305 PMC12300321 40731934

[16] Choi KY Lee SW In Y Kim MS Kim YD Lee SY Lee JW Koh IJ. Dual-energy CT-Based bone mineral density has practical value for osteoporosis screening around the knee. Medicina (Lithuania). 2022;58(8):1085. 10.3390/medicina58081085 PMC9416743 36013552

[17] Stentz-Olesen K Nielsen ET De Raedt S Jørgensen PB Sørensen OG Kaptein BL Andersen MS Stilling M. Validation of static and dynamic radiostereometric analysis of the knee joint using bone models from CT data. Bone Joint Res. 2017;6(6):376-84. 28600383 10.1302/2046-3758.66.BJR-2016-0113.R3 PMC5492337

[18] Forlenza EM Serino J Terhune EB Weintraub MT Nam D Della Valle CJ. Cementless total knee arthroplasty is associated with early aseptic loosening in a large national database. J Arthroplasty. 2023;38(7 suppl 2):S215-20. 10.1016/j.arth.2023.02.058 36863574

[19] Andronic O Yang YH Pabbruwe M Jones CW Yates PJ. Early aseptic loosening and inferior patient-reported outcomes of a cementless tibial baseplate in a modern total knee arthroplasty designCite this article. Bone Joint J. 2025;107(4):440-8. 40164184 10.1302/0301-620X.107B4.BJJ-2024-0704.R1

[20] Kohli N Stoddart JC van Arkel RJ. The limit of tolerable micromotion for implant osseointegration: a systematic review. Sci Rep. 2021;11(1):10797. 34031476 10.1038/s41598-021-90142-5 PMC8144379

[21] Andersen MR Winther N Lind T Schrøder H Flivik G Petersen MM. Tibial component undersizing is related to high degrees of implant migration following cementless total knee arthroplasty: a study of radiostereometric analysis data for 111 patients with 2-Year Follow-up. JBJS Open Access. 2023;8(3):e23:00032. 10.2106/JBJS.OA.23.00032 PMC10412432 37575962

[22] Gibian JT Zuke WA Hood H Blum E Nunley RM Barrack RL Bendich I. Early aseptic tibial loosening is a concern with a modern two-peg cementless total knee arthroplasty design. J Arthroplasty. 2025;40(3):678-82. 39307203 10.1016/j.arth.2024.09.023

[23] Nam D Lawrie CM Salih R Nahhas CR Barrack RL Nunley RM. Cemented versus cementless total knee arthroplasty of the same modern design: a prospective, randomized trial. J Bone Joint Surg Am. 2019;101(13):1185-92. 31274720 10.2106/JBJS.18.01162 PMC6641115

[24] Mikashima Y Imamura H Shirakawa Y Yano K Ikari K Okazaki K. Modern cementless posterior stabilized mobile-bearing total knee arthroplasty shows comparable clinical and radiographical results to its cemented predecessor at 1-year follow-up. Knee Surg Sports Traumatol Arthrosc. 2022;30(9):3131-7. 35781580 10.1007/s00167-022-07047-7

[25] Yao K Chen Y. Comprehensive evaluation of risk factors for aseptic loosening in cemented total knee arthroplasty: a systematic review and meta-analysis. J Exp Orthop. 2024;11(3):e12095. 39035847 10.1002/jeo2.12095 PMC11260281

[26] Ayati Firoozabadi M Mafi AH Afzal S Beheshti Fard S Khaledian H Bozorgsavoji A Azadnajafabad S Mortazavi SMJ. Does body mass index (BMI) significantly influence aseptic loosening in primary total knee arthroplasty? Insights from a long-term retrospective cohort study. BMC Musculoskelet Disord. 2024;25(1):980. 39616316 10.1186/s12891-024-07913-0 PMC11607931

[27] van Duren BH Firth AM Berber R Matar HE James PJ Bloch BV. Revision rates for aseptic loosening in the Obese patient: a comparison between stemmed, uncemented, and unstemmed tibial total knee arthroplasty components. Arthroplast Today. 2025;32:101621. 40083895 10.1016/j.artd.2025.101621 PMC11904599

[28] Mikashima Y Imamura H Shirakawa Y Yano K Takagi H Okazaki K. The vast majority of radiolucent lines disappeared at 3 years follow-up in modern cementless posterior stabilized mobile-bearing total knee arthroplasty. Arch Orthop Trauma Surg. 2025;145(1):265. 40274614 10.1007/s00402-025-05880-2

[29] Reina N Cavaignac E Pailhé R Pailliser A Bonnevialle N Swider P Laffosse JM. BMI-related microstructural changes in the tibial subchondral trabecular bone of patients with knee osteoarthritis. J Orthop Res. 2017;35(8):1653-60. 27747928 10.1002/jor.23459

[30] Sun C Wang C Li J Liu C Wei Z Bi Z Li Y Li S. Creating perforations in the sclerotic region of the proximal tibia during total knee arthroplasty to enhance prosthesis stability. Orthop Surg. 2025;17(5):1397-405. 40104929 10.1111/os.70025 PMC12050191

[31] Itou J Kuwasawa A Nihei K Okazaki K. Proximal tibia bone mineral density correlates more closely with hip density in men with knee osteoarthritis. J Joint Surg Res. 2025;3(3):121-7.

[32] Ministry of Health, Labour and Welfare. Medical Fee Schedule Database; 2026. https://www.mhlw.go.jp/stf/seisakunitsuite/bunya/0000198757.html. Accessed April 25, 2026.

[33] Kobayashi M Asada Y Matsubara K Haba T Matsunaga Y Kawaguchi A Katada K Toyama H Koshida K Kato R Suzuki S. Evaluation of effective dose using the k-Factor of optimal scan range for CT examination. Open J Radiol. 2015;05(03):172-48.

[34] RadiologyInfo.org. Radiation Dose from X-Ray and CT Exams; 2026. https://www.radiologyinfo.org/en/info/safety-xray. Accessed April 25, 2026.

[35] Krishnan S Li D Mao S Flores F Gao Y Luo S Budoff M. Comprehensive assessment of CT hounsfield unit variation within patients, scanners, and integrated measures. J Cardiovasc Computed Tomography. 2024;18(4):S64.

[36] Dudle A Ith M Egli R Heverhagen J Gugler Y Wapp C Frauchiger DA Lippuner K Jackowski C Zysset P. Asynchronous calibration of a CT scanner for bone mineral density estimation: sources of error and correction. JBMR Plus. 2024;8(9):ziae096. 39183821 10.1093/jbmrpl/ziae096 PMC11344033

[37] Piscone S Saccone S Milillo P Schiraldi G Vinci R Macarini L Stoppino LP. Bone mineral density (BMD) assessment using dual-energy CT with different base material pairs (BMPs). J Imaging. 2025;11(7):236. 40710622 10.3390/jimaging11070236 PMC12295864

[38] Sato A Ota M Miyazawa T Takizawa M Nagasaka R Mukunoki M Izukashi K Oike J Okumo T Yagura S Koya T Kanzaki K. Intraoperative evaluation of bone resection accuracy in total knee arthroplasty using an augmented reality-based navigation system. Arch Orthop Trauma Surg. 2025;145(1):414. 40833637 10.1007/s00402-025-06033-1

[39] Petrillo S Migliorini F Moretti G Romagnoli S. Accuracy of ROSA knee system in bone cuts orientation during total knee arthroplasty: an observational study. J Clin Med. 2025;14(15):5205. 40806827 10.3390/jcm14155205 PMC12347779

[40] Baek JH Lee SC Lee DN Heo J Kim T Ahn HS Nam CH. Better accuracy of robotic-assisted total knee arthroplasty compared to conventional technique in patients with failed high tibial osteotomy. PLoS One. 2024;19(11):e0313391. 39527607 10.1371/journal.pone.0313391 PMC11554210

[41] Leong JWK Lee BH Haniball J Thwin L Kunnasegaran R Tan KG. Retrospective analysis of early functional and radiological outcomes between robotic, navigated and conventional total knee arthroplasty: a single Surgeon's series. J Clin Orthop Trauma. 2025;69:103145. 40838090 10.1016/j.jcot.2025.103145 PMC12362120

[42] Ghossein A Gatz M Dohmen A Hildebrand F Hofmann UK. Use of robotics may facilitate earlier functional recovery and reduce overcorrection compared to conventional implantation techniques in total knee arthroplasty: a single-surgeon cohort study. BMC Musculoskelet Disord. 2025;26(1):992. 41131523 10.1186/s12891-025-09102-z PMC12548222

[43] Montagna A Marescalchi M Andriollo L Sangletti R Benazzo F Rossi SMP. Robotics in TKA: state of the art and future perspectives. Joints. 2025;3:e1523.

[44] Garibaldi R Lustig S Vella-Baldacchino M Fiore PI Batailler C. Prevalence of low bone mineral density in robotic-assisted TKA candidates: insights from quantitative CT analysis. SICOT J. 2025;11:57. 41172241 10.1051/sicotj/2025048 PMC12578436

[45] Tigani D Lamattina L Puteo N Donadono C Banci L Colombo M Pizzo A Assenza A. Novel clinical applications of 3D-printed highly porous titanium for off-the-shelf cementless joint replacement prostheses. Biomimetics. 2025;10(9):634. 41002868 10.3390/biomimetics10090634 PMC12467573

